# A Stochastic Approach to Noise Modeling for Barometric Altimeters

**DOI:** 10.3390/s131115692

**Published:** 2013-11-18

**Authors:** Angelo Maria Sabatini, Vincenzo Genovese

**Affiliations:** The BioRobotics Institute, Scuola Superiore Sant'Anna/P.zza Martiri della Libertà, 33, 56124 Pisa, Italy; E-Mail: vincenzo.genovese@sssup.it

**Keywords:** barometric altimeters, stochastic modeling, system identification, motion tracking

## Abstract

The question whether barometric altimeters can be applied to accurately track human motions is still debated, since their measurement performance are rather poor due to either coarse resolution or drifting behavior problems. As a step toward accurate short-time tracking of changes in height (up to few minutes), we develop a stochastic model that attempts to capture some statistical properties of the barometric altimeter noise. The barometric altimeter noise is decomposed in three components with different physical origin and properties: a deterministic time-varying mean, mainly correlated with global environment changes, and a first-order Gauss-Markov (GM) random process, mainly accounting for short-term, local environment changes, the effects of which are prominent, respectively, for long-time and short-time motion tracking; an uncorrelated random process, mainly due to wideband electronic noise, including quantization noise. Autoregressive-moving average (ARMA) system identification techniques are used to capture the correlation structure of the piecewise stationary GM component, and to estimate its standard deviation, together with the standard deviation of the uncorrelated component. *M*-point moving average filters used alone or in combination with whitening filters learnt from ARMA model parameters are further tested in few dynamic motion experiments and discussed for their capability of short-time tracking small-amplitude, low-frequency motions.

## 1. Introduction

Barometric altimeters can be used for measuring the height of an object above a given reference level, e.g., the sea level—a popularly used term for this height is pressure altitude. Common applications are, e.g., in avionics, where barometric altimeters can help in stabilizing the vertical position and velocity of a flying object [1,2]. Recently, barometric altimeters have been considered for interesting human-centric applications, such as personal navigation and human motion tracking and monitoring. Beside their use as wristop computers for leisure and sports, barometric altimeters were integrated within multi-sensor pedestrian navigation systems and activity monitors, for improving performance of dead-reckoning algorithms or classifiers of human motion patterns: e.g., they were used to detect the moving styles of going up/down the stairs or in an elevator [3]; to determine the correct floor of a user in a multi-storey building [4]; to detect stair ascent-descent in ambulatory monitors designed for estimating the energy expenditure incurred during activities of daily living, including stair-walking [5,6]. More recently, it was proposed that the measured pressure altitudes might help improving accelerometry-based fall detection systems [7–10]. The rationale behind this application is that, when a person is standing, the difference in atmospheric air pressure between the waist and the feet can be monitored for sudden changes that would indicate a fall event [7]. However, rapid changes in pressure uncorrelated with altitude can be generated, outdoors, by unpredictable atmospheric conditions, and, indoors, by frequent opening/closing of windows or doors and by air conditioning systems [11]. The consequence is that barometric altimeter data tends to be very noisy, and their accuracy is rather poor.

Recent advances in MEMS sensing technologies have led to the availability of barometric altimeters that are well suited for integration in mobile and portable systems. The most popular barometric altimeters for human-centric applications are manufactured by Bosch Sensortec (Reutlingen, Germany) [8–12] and VTI Technologies (Vantaa, Finland) [6,7,9]. These sensors can be used in few measurement modes, which differ in the resolution offered for a given sampling rate. Published recipes for pressure altitude signal conditioning and pre-processing do not go beyond generic prescriptions for low-pass filtering, e.g., in order to obtain an accuracy of 0.1 m using the barometric altimeter alone, several seconds of averaging would be necessary [12].

In this paper we develop a method for capturing salient statistical properties of the noise that affects barometric altimeters (stochastic approach to noise modeling). Using the developed model we intend to assess the short-time tracking performance of small-amplitude, low-frequency motions. By the term short-time motion tracking we refer to motions with durations up to few minutes, in which relative changes of altitude are to be tracked. Conversely, long-time motion tracking involves accurate tracking of absolute altitude over long time intervals. In human-centric applications, both tracking modes are involved: short-time tracking, e.g., fall detection; long-time tracking, e.g., personal navigation. Under conditions of short-time motion tracking, the time-varying mean is expected to vary very slowly, contributing an offset in the measured pressure altitude with no practical effect on the tracking accuracy. In those applications where accurate absolute altitude is needed, barometric altimeters in differential mode are perhaps the best option available to deal with slow changes of atmospheric pressure [11].

The proposed method of analysis decomposes the noise in three additive components with different physical origin and properties: a deterministic time-varying mean, correlated with slow pressure changes that can be involved in, e.g., local weather forecasting [13] and long-time tracking [14], whose modeling is beyond the scope of this paper; an exponentially time-correlated random process, modeled as a first-order Gauss-Markov (GM) process, that accounts for short-term, local environment changes, whose effect is prominent for short-time tracking; an uncorrelated random process, mainly due to wideband electronic noise, including quantization noise. We take the novel approach of using Gauss Markov (GM) random processes for modeling the short-time correlated component of altimeter noise. The reason for this choice is that GM random processes are mathematically simple, with only a limited number of parameters that need to be estimated [15]. Moreover, they seem to fit a wide variety of physical phenomena with reasonable accuracy [16]. Autoregressive-moving average (ARMA) system identification techniques are used to capture the correlation structure of the piecewise stationary GM component, and to estimate its standard deviation, together with the standard deviation of the uncorrelated component.

Experimental results obtained when the barometric altimeter is either motionless (for model identification) or moving (for tracking performance assessment) are presented. To this aim *M*-point moving average filters are applied to time-correlated pressure altitude signals, alone or cascaded with whitening filters learnt from ARMA model parameters. Advantages and disadvantages of removing the short-term correlation of the GM component by whitening filters are discussed.

## 2. Methods

### 2.1. Measurement Process

The International Standard Atmosphere (ISA) model can be used to calculate the altitude from the output of an air pressure sensor [17]. The basic physical equations used for modeling the atmosphere are the hydrostatic equation:

(1) $$\frac{d}{dh}p=-\rho g$$

and the ideal gas law:

(2) $$p=\rho \bar{R}T,$$

where *h* is the altitude, *p* is the air pressure, *ρ* is the average density of the air, *g* is the gravity acceleration (*g* = 9.80665 m/s^2^), *T* is the absolute temperature and *R̅* is the specific gas constant (*R̅* = 287.058 J·kg·K^−1^, in conditions of dry air). The ISA model prescribes that the standard pressure at sea level is *p*_o_ = 1,013.25 hPa, and the standard temperature is *T*_o_ = 15 °C (288.15 K).

Equations (1) and (2) can be combined together yielding the first-order differential equation:

(3) $$\frac{d}{dh}p=-\frac{g}{\bar{R}T}p.$$

The assumption of constant gravity is not crucial in solving Equation (3), that is, the variation of gravity with altitude and latitude can be safely ignored for short-distance trips [18]. Moreover, it is known that temperature tends to change with altitude. The lapse rate is defined as the rate of temperature increase in the atmosphere with the altitude: a constant lapse rate *L* can be assumed between 0 and 11 km (*L* = −6.5 K/km)—the negative sign indicates that the temperature decreases with altitude.

Equation (3) can be solved under the assumption of constant gravity and lapse rate, yielding the barometric formula:

(4) $$h=-\frac{T_{o}}{L}\left(1-{\left(\frac{p}{p_{o}}\right)}^{-\frac{L\bar{R}}{g}}\right)=44.300\left(1-{\left(\frac{p}{p_{o}}\right)}^{0.19}\right),$$

where *h* is expressed in m.

The ISA model fails to accurately describe the real atmosphere in many ways. The assumption of hydrostatic equilibrium is generally valid, provided that the effects of short-term winds can be tolerated. In the real atmosphere significant variations are also observed in pressure, temperature and even lapse rate. Moreover, although the ideal gas assumption is highly accurate for air, the behavior of an ideal gas is influenced by *R̅* the value of which depends in turn on mean molecular weight. The composition of the lower atmosphere is approximately constant, but in a very wet atmosphere the water vapor content can be high enough to significantly lower the density of the air, thus changing the value of *R̅*.

Absolute altitude information cannot be easily obtained; in particular, it is necessary to know, *inter alia*, the local sea level pressure, which may differ from standard pressure. Equation (4) still applies in cases where the pressure and the temperature differ from those of the standard: the relative change in pressure and the actual temperature determine the change in altitude regardless of altitude. Fortunately, it is the change in altitude to be important in, e.g., human-centric applications including fall detection. To overcome difficulties in matching the ISA model to real atmospheric conditions, the use of a reference barometric altimeter at a known and constant altitude has been also proposed to obtain altitude from a moving barometric altimeter (differential barometry), although local disturbances in pressure were critical for achieving high accuracy [11].

### 2.2. Stochastic Approach to Noise Modeling

**Figure 1.**
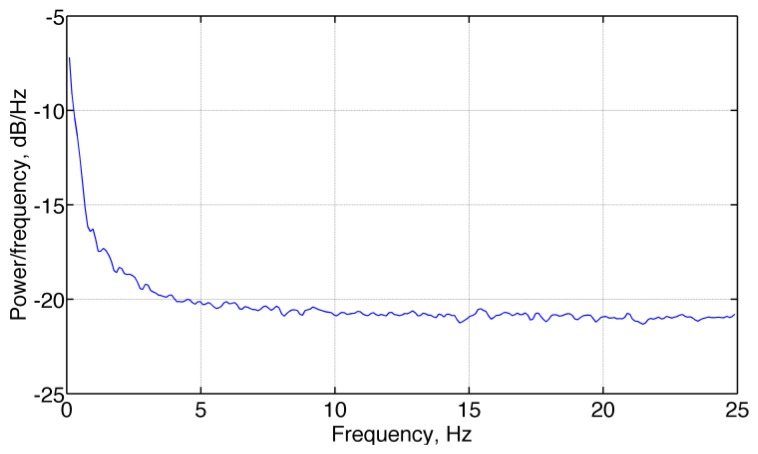
Pressure altitude noise spectrum from a barometric altimeter.

Figure 1 shows a typical portrait of the spectrum of barometric altimeter noise (pressure altitude). The concentration of noise energy in the low frequency region of the spectrum (colored noise) can be explained as the consequence of, e.g., thermal gradients and air flow in the sensor vicinity.

In this paper the noise *n*(*t*) superimposed to the output of a barometric altimeter is assumed to be the additive combination of three different components:

(5) $$n(t)=n_{c}(t)+n_{u}(t)+b(t).$$

where *b*(*t*) is the slowly time-varying mean, of no interest to us here, since our focus is in short-time tracking applications −*b*(*t*) was modeled as a GM random process for the long-time tracking simulations reported in [14], where model identification from experimental data was not attempted; the correlated noise component *n_c_*(*t*) is modeled as a short-term stationary GM stochastic process with zero mean, variance $\sigma_{c}^{2}$ and exponential auto-correlation function with correlation time constant *τ*, the uncorrelated noise component *n_u_*(*t*) is modeled as a white Gaussian stochastic process with zero mean and variance $\sigma_{u}^{2}$.

*n_c_*(*t*) is produced from passing a white Gaussian driving noise with zero-mean and unit variance through a first-order shaping filter with frequency response:

(6) $$H_{c}(\omega )=\frac{\beta }{1+j\omega \tau }.$$

The standard deviation σ*_c_* at the output of the shaping filter Equation (6) is given by:

(7) $$\sigma_{c}=\frac{\beta }{\sqrt{2\tau }}.$$

*n_u_*(*t*) can be described in a similar fashion. The zero-order shaping filter can be approximated over the sensor bandwidth as follows:

(8) $$H_{u}(\omega )=\alpha .$$

The standard deviation *σ_u_* is therefore given by:

(9) $$\sigma_{u}=\alpha .$$

Applying the spectral factorization technique to the additive combination of the spectral densities of *n_c_*(*t*) and *n_u_*(*t*), which are assumed independent, the following expression of the overall shaping filter is obtained:

(10) $$H(\omega )=\sqrt{\alpha^{2}+\beta^{2}}\frac{1+j\omega \tau_{o}}{1+j\omega \tau_{p}},$$

where:

(11) $$\{\begin{array}{l} \tau_{p}=\tau \\ \tau_{o}=\frac{\alpha }{\sqrt{\alpha^{2}+\beta^{2}}} \end{array}$$

Since the pressure altitude samples from the barometric altimeter are available only at discrete times that are integer multiples of the sampling interval *T_s_*, the shaping filter Equation (10) is discretized using the bilinear transform technique, yielding:

(12) $$H(z^{-1})=H_{o}\frac{1+bz^{-1}}{1+az^{-1}},$$

where:

(13) $$H_{o}=\sqrt{\alpha^{2}+\beta^{2}}\frac{1+\frac{2\tau_{o}}{T_{s}}}{1+\frac{2\tau_{p}}{T_{s}}}$$

and:

(14) $$\{\begin{array}{c} b=\frac{1-\frac{2\tau_{o}}{T_{s}}}{1+\frac{2\tau_{o}}{T_{s}}}\\ a=\frac{1-\frac{2\tau_{p}}{T_{s}}}{1+\frac{2\tau_{p}}{T_{s}}} \end{array}$$

Equations (7), (9), (11), (13), (14) allow us to compute *σ_c_*, *σ_u_* and *τ* from *H_o_*, *a*, and *b*. For example, the correlation time constant is given by:

(15) $$\tau =\frac{T_{s}}{2}\frac{1-a}{1+a}.$$

Using autoregressive moving average (ARMA) system identification techniques, *H_o_*, *a*, and *b* can be estimated from air pressure signals that were previously detrended by subtracting the time-varying mean.

Under the assumption that the shaping filter Equation (12) is identified using the ARMA(1,1) model structure and the prediction-error approach to parameter estimation [15], the asymptotic covariance matrix of the estimates *a_e_* and *b_e_* of the parameters *a* and *b* is given by [19]:

(16) $$C\approx \frac{1}{N}\frac{1-a_{e}b_{e}}{{\left(a_{e}-b_{e}\right)}^{2}}\left[\begin{array}{cc} \left(1-a_{e}^{2})(1-a_{e}b_{e}\right) & \left(1-a_{e}^{2})(1-b_{e}^{2}\right)\\ \left(1-a_{e}^{2})(1-b_{e}^{2}\right) & \left(1-b_{e}^{2})(1-a_{e}b_{e}\right) \end{array}\right]$$

The estimates returned by the prediction-error approach are known to be asymptotically normally distributed as the number *N* of data samples tends to infinity. However, *σ_c_*, *σ_u_* and *τ* are non-linearly related to *H_o_*, *a*, and *b*. Departures from Gaussianity can then be observed in the distributions of the estimates of *σ_c_*, *σ_u_* and for any finite *N*. The problem is especially acute for the correlation time constant: not surprisingly, since estimating the correlation time constant of a GM random process is known to require a relatively huge amount of data for accurate results [20].

The experimental distribution of *τ* turns out to be right-skewed, since the estimated value of the model pole is close to the unit circle. For a given observation period, as needed to preserve the short-term stationarity, the attempts to improve the estimation accuracy by increasing *N* are counterweighted by the decrease of the sampling interval, with a consequent shift of the model pole toward the critical point *z* = 1.

### 2.3. Sensor Hardware

The experiments described in this paper were performed using a battery-powered wearable inertial measurement unit (WIMU) whose development is undergoing in our lab for applications in human motion ambulatory monitoring and assessment. The WIMU is endowed with a 32-bit ARM Cortex processor (LPC1768, NXP Semiconductors, Eindhoven, the Netherlands) and a Bluetooth transceiver for data communication with an Android-based smart-phone (Samsung Galaxy SII, GT-I9100, Samsung, Seoul, South Korea). The WIMU sensors are a digital tri-axial gyro (ITG-3200, InvenSense, San Jose, CA, USA), a digital tri-axial accelerometer (BMA180, Bosch, Reutlingen, Germany), a digitaltri-axial magnetic sensor (HMC5843, Honeywell, Morristown, NJ, USA), and a digital pressure sensor (Bosch BMP085).

The measurements by the BMP085 digital pressure sensor are noisy, including a significant amount of quantization noise due to a coarse resolution of about 1 Pa (corresponding to about 8.43 cm). The BMP085 was used in the ultra-low power mode (no oversampling was performed internally to the sensor). The ANSI C code by the sensor manufacturer was used to implement the compensation algorithm for pressure and temperature measurement using the integrated thermal sensor [21]. We ported this piece of software to the ARM controller of the WIMU, together with the routine for calculating the altitude from calibrated pressure measurements using Equation (4). The temperature was measured once per second, and this value was used for all pressure altitude measurements during the same period. Pressure altitude data were computed by the ARM controller of the WIMU and logged to the smart-phone at a rate of 50 Hz, before their upload to a PC for subsequent analysis.

### 2.4. Experiments

The barometric altimeter was tested in static and motion conditions. The barometric altimeter was motionless on a table while six data acquisition trials, each of one lasting 10 min, were performed in different day hours, either in a household environment or outdoors (tests in static conditions). The testing procedure was repeated a second time, days later, in similar environmental conditions as above. Static tests allowed ARMA model identification and validation. Based on the ARMA model parameters, a whitening filter was also designed by inverting and discretizing the shaping filter Equation (10), as explained in the next subsection.

Two motion tracking experiments were also performed (tests in motion conditions). These experiments with a moving barometric altimeter were used to test few methods of signal processing: Method A (no filtering); Method B (4-point moving average filtering); Method C (4-point moving average filtering cascaded with the designed whitening filter). In the first experiment, the WIMU was mounted at the waist of a person who walked indoors along stairs spanning 1.7 m for each floor in a series of three distinct floors from ground level (*stair-walking*). During the second motion tracking experiment (*forced circular motion*), the WIMU was affixed at the distal part of a plastic rod; the rod was hinged on the axis of an electric motor controlled in rotation speed using a user interface running on a PC. The rod was lying in a plane aligned to gravity, so as the barometric altimeter traced a circle of radius 30 cm; the rod was rotated for two minutes at each of four different motion frequencies, 0.25, 0.5, 1, and 1.5 Hz. The measurement data (pressure altitude from the barometric altimeter and rod angular position by the incremental encoder fitted to the motor axis) were electrically synchronized and logged to the host computer for further analysis. The vertical position of the barometric altimeter relative to its initial position was estimated using the encoder measurements and the known length of the rod, yielding the reference against which the relative change in pressure altitude estimated by the barometric altimeter was compared for Root Mean Square Error (RMSE) analysis.

**Figure 2.**
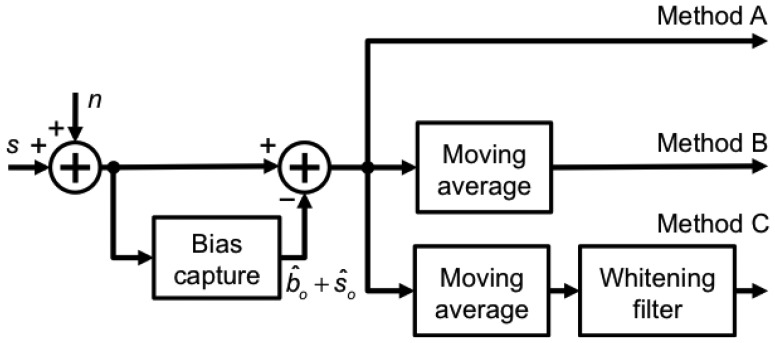
Tested methods of signal processing.

During motion tracking experiments, the relative pressure altitude was computed by taking the average value of the absolute pressure altitude *b̂_o_* + *ŝ_o_* during a rest period of 1 s before the motion started; the absolute pressure altitude signals were then detrended by subtracting this constant value from them (bias capture), Figure 2.

### 2.5. Data Processing for ARMA System Identification

**Figure 3.**
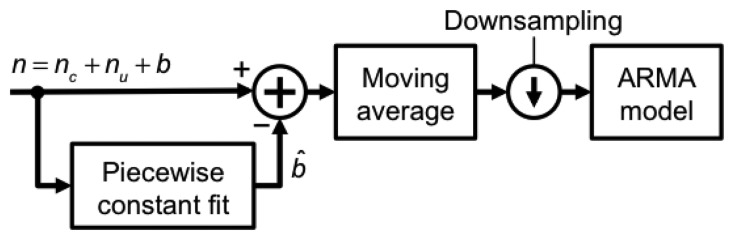
Pressure altitude noise pre-processing (modeling task).

Data processing was carried out using MATLAB. Piecewise constant fitting of raw data from static tests was performed using data windows of fixed size (1 min), and data were locally detrended by subtracting the constant level (detrended raw data). After applying a 4-point moving average filter, the filtered data were downsampled by 5 (*T_s_* = 0.1 s), in preparation for ARMA system identification, Figure 3.

ARMA model parameters were then estimated for each non-overlapping data window of *N* = 300 data samples (*i.e.*, 30 s) using the prediction-error approach. The adequacy of the fitted model was assessed applying the Bartlett's test of whiteness to the prediction-error sequence at the 95% level. The Akaike's final prediction error criterion was used to defend the ARMA(1,1) model against higher-order models.

The sampling interval and the window length were chosen with the aim to reduce the model parameter uncertainty while preserving the short-term stationarity of the processed data. The short-term stationarity was assessed using the spectral error measure approach [15]. The model parameters values were averaged (10% trimmed mean) and the standard deviation (SD) was computed on the trimmed values—the reason for trimming was to reject outliers that were observed, especially when the correlation time constant was estimated. Once the model was successfully identified and validated, the shaping filter Equation (10) was inverted and discretized using the bilinear transform technique at the sampling frequency of 50 Hz (whitening filter). The detrended raw data were then filtered using the 4-point moving average cascaded with the whitening filter and the procedure of model identification was applied to time-decimated filtered data. In principle, the whitening filter generates surrogate data where the serial correlation present in the input data is removed. When ARMA system identification was applied to surrogate data, zero-pole cancellation was verified by testing the null hypothesis that the difference between *a_e_* and *b_e_* was null (paired sample *t*-test, *p* > 0.1). The standard deviation of the uncorrelated noise component was computed as *σ_u_* = *H_o_*.

## 3. Results

### 3.1. ARMA Model Identification and Validation

Tables 1 and 2 report the 10% trimmed mean value ± SD of *σ_c_*, *σ_u_* and *τ* for the experiments in static conditions of the first and second day, respectively. The last column gives the total standard deviation *σ_s_* of the pressure altitude signals.

**Figure 4.**
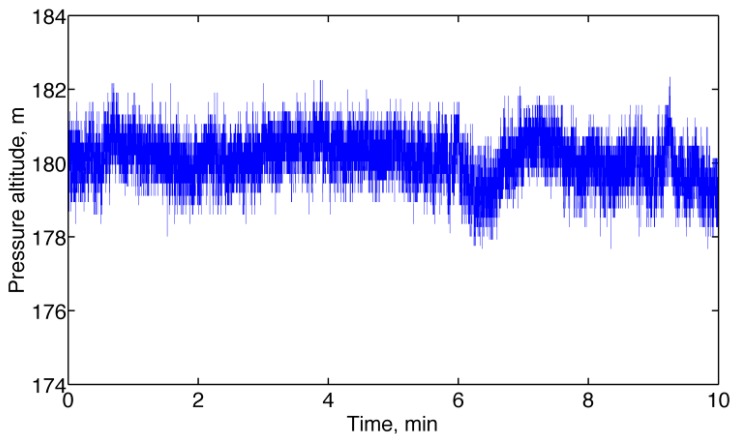
Pressure altitude recorded from the barometric altimeter (outdoors static test).

The standard deviation of the correlated noise component took higher values than in relatively unperturbed conditions, while the standard deviation of the uncorrelated noise component was relatively unchanged. Figure 4 reports a representative plot of measured pressure altitude.

A one-way analysis of variance was performed for comparing the means of the six groups of point estimates of *a* and *b* from the experiments during the first day. The null hypothesis that the means of the groups were equal was accepted, yielding: *a_e_* = −0.86 ± 0.03 (*p* > 0.4), and *b_e_* = −0.52 ± 0.06 (*p* > 0.26)—the agreement with the estimation uncertainty computed theoretically from Equation (16) was also very good. The whitening filter designed using these point estimates had a pole at a frequency of about 1 Hz, a zero approximately two octave down; a unity-gain constraint was enforced at 25 Hz, yielding a DC gain of about 0.21.

Surrogate data were generated by applying the whitening filter to the detrended raw data from both days. Surrogate data were submitted to ARMA identification after 4-point moving average filtering and time decimation, as described above. Zero-pole cancellation took place, even when the whitening filter was applied to data from the second day, which means that the whitening filter was effective in removing serial correlation from surrogate data, Tables 3 and 4. The last column gives the total standard deviation *σ_s_* of the whitened pressure altitude signals.

**Figure 5.**
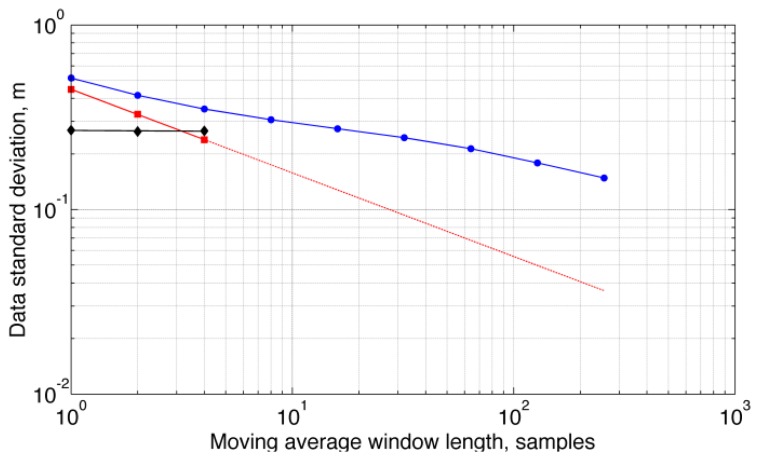
Total standard deviation (●), standard deviation of the correlated (♦) and uncorrelated (▪) noise components. The line superimposed to the values of the standard deviation of the uncorrelated noise component indicates the theoretical scaling with the number of samples involved in the averaging process.

Detrended raw data from static tests were also submitted to ARMA identification when *M*-point moving average filtering was applied for dyadic values of *M*, Figure 5. Due to frequent failures of the Bartlett's test of whiteness, the ARMA(1,1) model was unable to fit the data for *M* ≥ 8.

### 3.2. Motion Tracking Tests

**Figure 6.**
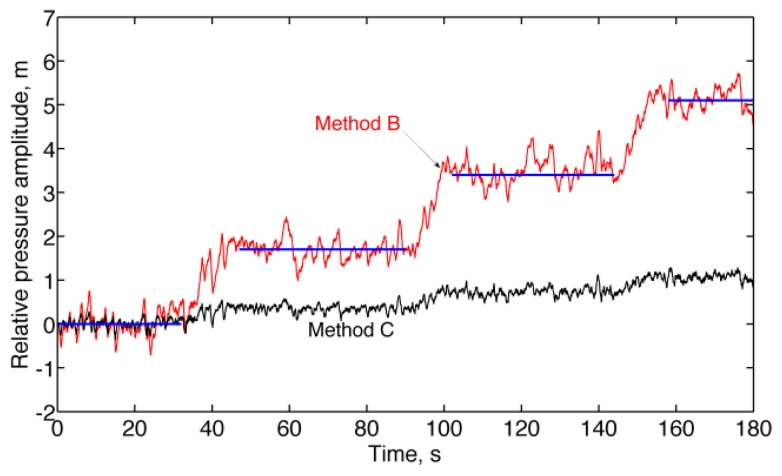
*Stair-walking* experiment. Transitions to higher-level floors from the ground floor are visible in the relative pressure altitudes estimated by Method B and Method C. Horizontal lines indicate the reference height of the floors relative to ground floor.

**Figure 7.**
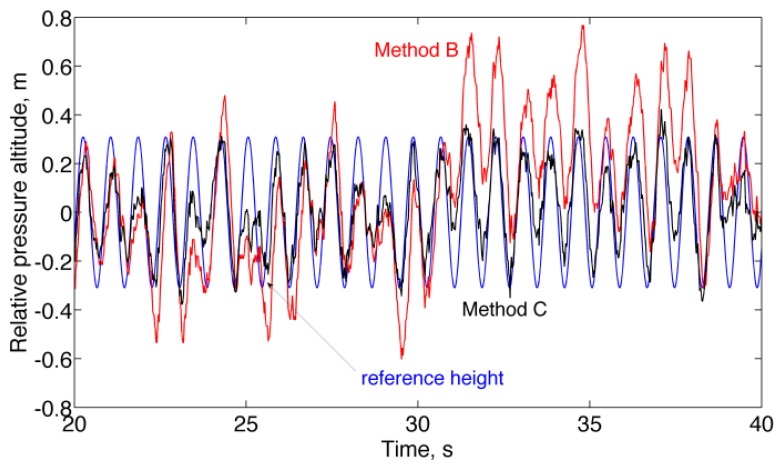
*Forced circular motion* experiment. The reference height is superimposed to the relative pressure altitudes estimated by Method B and Method C.

Finally, the results of the experiments in motion conditions are presented in Figure 6 and Table 5 (*stair-walking*), Figure 7 and Table 6 (*forced circular motion*). For better readability of the plots, the raw relative pressure altitudes (Method A) are not reported in Figures 6 and 7.

## 4. Discussion

One important result from using the method of analysis developed in this paper is that the generic prescription that several seconds of averaging of barometric altimeter data are necessary to achieve accuracy in the order of 0.1 m is quite misleading. The correlated noise component should be accounted when assessing the benefits of averaging. In all performed static tests, either indoors or outdoors, the correlated noise component turned out to have a greater standard deviation than the uncorrelated noise component, Tables 1 and 2, raising concerns about the suitability of, e.g., moving average filters to reduce altimeter noise.

The correlated noise component was virtually unaffected by an *M*-point moving average filter when *M* ≥ 4 at a sampling frequency of 50 Hz, and its standard deviation would decrease very slowly for higher values of *M*, although it was not possible to estimate the ARMA(1,1) model parameters for *M* ≥ 8, Figure 5. This was due to the fact that data fitting would include the *M*-point moving average filter itself, which called for higher-order ARMA models. The behavior of the total standard deviation shows that the $1\sqrt{M}$ scaling law, valid for uncorrelated data, is optimistic when dealing with time-correlated data [22]. A noise level of 0.15 cm was reached only when *M* = 256, which corresponds to an averaging time of about 5 s and a delay of 2.5 s in the filtering chain; on the other hand, *M* = 32 would suffice to reach a noise level of 0.1 cm if only uncorrelated noise would be present in the barometric altimeter output (however, *M* = 32 is equivalent to a delay in the filtering chain of 320 ms that can be unacceptably high in several human-centric applications, e.g., pre-impact fall detection [23]).

The whitening filter was a highly effective tool for filtering the correlated noise component, as shown by the results reported in Tables 3 and 4. However, a problem with its use in dynamic motion experiments was related to where the unity-gain constraint was enforced. Since the correlated noise component occupies the low-frequency part of the spectrum, the whitening filter presents a typically high-pass frequency response, which tends to amplify uncorrelated noise unless the unity-gain constraint is moved from DC to higher frequencies. Accurate height tracking would then be possible for dynamic motion frequencies exceeding, say, few Hertz; unfortunately, typical vertical human motions, such as walking up or down a staircase, have motion frequencies much smaller than the −3 dB corner frequency of the whitening filter. The amplifying effect on the uncorrelated noise component due to a unity-gain constraint enforced at DC would require, approximately, a 25-fold increase in the length of the *M*-point moving average filter for achieving the same Signal-to-Noise ratio, which is untenable in practice.

The results of the *stair-walking* experiment clearly show the difficulty of placing the unity-gain constraint at high frequency (Figure 6 and Table 5). The pressure altitude change from one floor to the next floor was underestimated, on average, by a factor close to the whitening filter DC gain when Method C was used, as compared to Method B, at the expense of much smaller fluctuations around the mean value. The advantages of using the whitening filter can be seen when analyzing the results of the *forced circular motion* experiment (Figure 7 and Table 6), in which the motion frequencies were higher than in the *stair-walking* experiment.

## 5. Conclusions

In this paper we have developed a method of analysis of barometric altimeter noise based on system identification techniques. Beside the slowly time-varying mean, which was outside our scope in the present context, the noise-like fluctuations on barometric altimeter output were interpreted as the superposition of a GM random process that explained short-term temperature and pressure changes (environment-dependent) and an uncorrelated noise component with relatively time-invariant statistics, which accounted for electronics noise. Based on the ARMA model parameters, a whitening filter was also designed and tested, together with standard *M*-point moving average filters, for improving short-time tracking of small-amplitude, low-frequency motions.

Because of their limited accuracy, it is presently a matter of debate whether barometric altimeters can be effectively used in human-centric applications, e.g., fall detection. It is likely that they will find their role as aiding sensors in systems where multi-sensor fusion methods for motion tracking and event detection are implemented. Oftentimes, these methods are optimal when the sensor noise is white, and their robustness may be an issue when the whiteness assumption is not valid [24]. The developed method can thus be used in building simulation environments for algorithm testing, as well as in providing practical tools, e.g., whitening filters, by means of which the performance of a given signal processing method can be improved. Moreover, the developed method can be used to perform a sort of environment probing, namely local environmental effects can be directly gauged in terms of the percentage of the total variance due to noise in barometric altimeters.

## Figures and Tables

**Table 1.** ARMA model parameters (10% trimmed mean ± SD)—first day.

	***τ*, s**	***σ_c_*, m**	***σ_u_*, m**	***σ_s_*, m**
Indoors static tests				
1	0.71 ± 0.27	0.26 ± 0.03	0.23 ± 0.01	0.33 ± 0.03
2	0.87 ± 0.34	0.27 ± 0.04	0.24 ± 0.01	0.34 ± 0.03
3	0.72 ± 0.21	0.26 ± 0.02	0.23 ± 0.01	0.33 ± 0.02

Outdoors static tests				
1	0.77 ± 0.30	0.26 ± 0.03	0.24 ± 0.01	0.34 ± 0.02
2	0.68 ± 0.32	0.29 ± 0.02	0.24 ± 0.01	0.36 ± 0.04
3	0.65 ± 0.23	0.27 ± 0.03	0.23 ± 0.01	0.34 ± 0.03

**Table 2.** ARMA model parameters (10% trimmed mean ± SD)—second day.

	***τ*, s**	***σ_c_*, m**	***σ_u_*, m**	***σ_s_*, m**
Indoors static tests				
1	0.69 ± 0.32	0.27 ± 0.04	0.24 ± 0.01	0.35 ± 0.04
2	0.82 ± 0.23	0.27 ± 0.05	0.25 ± 0.01	0.35 ± 0.04
3	0.78 ± 0.36	0.29 ± 0.05	0.27 ± 0.01	0.38 ± 0.05

Outdoors static tests				
1	0.75 ± 0.34	0.24 ± 0.03	0.22 ± 0.01	0.32 ± 0.03
2	1.26 ± 0.39	0.29 ± 0.03	0.23 ± 0.01	0.34 ± 0.04
3	0.98 ± 0.26	0.28 ± 0.04	0.25 ± 0.03	0.36 ± 0.05

**Table 3.** ARMA model parameters (10% trimmed mean ± SD)—surrogate data, first day.

	***τ*, s**	***σ_c_*, m**	***σ_u_*, m**	***σ_s_*, m**
Indoors static tests				
1	--	--	0.22 ± 0.02	0.24 ± 0.01
2	--	--	0.23 ± 0.02	0.24 ± 0.01
3	--	--	0.22 ± 0.02	0.23 ± 0.01

Outdoors static tests				
1	--	--	0.22 ± 0.03	0.25 ± 0.01
2	--	--	0.23 ± 0.02	0.25 ± 0.01
3	--	--	0.21 ± 0.03	0.24 ± 0.01

**Table 4.** ARMA model parameters (10% trimmed mean ± SD)—surrogate data, second day.

	***τ*, s**	***σ_c_*, m**	***σ_u_*, m**	***σ_s_*, m**
Indoors static tests				
1	--	--	0.23 ± 0.02	0.25 ± 0.01
2	--	--	0.24 ± 0.01	0.25 ± 0.02
3	--	--	0.26 ± 0.04	0.27 ± 0.02

Outdoors				
1	--	--	0.21 ± 0.03	0.22 ± 0.01
2	--	--	0.20 ± 0.03	0.23 ± 0.01
3	--	--	0.25 ± 0.03	0.25 ± 0.03

**Table 5.** Mean value ± SD of the pressure altitude, expressed in meters. Statistics computed using signal plateaus for each floor, including ground floor (*stair-walking* experiment).

	**Ground Floor**	**Floor #1**	**Floor #2**	**Floor #3**
Reference	0.00	1.70	3.40	5.10
Method A	0.32 ± 0.53	1.99 ± 0.49	3.97 ± 0.53	5.39 ± 0.53
Method B	0.31 ± 0.31	1.99 ± 0.27	3.80 ± 0.33	5.41 ± 0.28
Method C	0.07 ± 0.15	0.41 ± 0.12	0.79 ± 0.14	1.12 ± 0.12

**Table 6.** RMSE between the pressure altitude and the reference vertical position, expressed in meters, for different values of motion frequency (*forced circular motion* experiment).

	**0.5 Hz**	**1 Hz**	**1.25 Hz**	**1.5 Hz**
Method A	0.53	0.53	0.50	0.52
Method B	0.34	0.35	0.32	0.34
Method C	0.20	0.18	0.18	0.19
